# GlyLES: Grammar-based Parsing of Glycans from IUPAC-condensed to SMILES

**DOI:** 10.1186/s13321-023-00704-0

**Published:** 2023-03-23

**Authors:** Roman Joeres, Daniel Bojar, Olga V. Kalinina

**Affiliations:** 1 Helmholtz Institute for Pharmaceutical Research Saarland (HIPS), Helmholtz Centre for Infection Research (HZI), Saarbruecken, Germany; 2 Center for Bioinformatics, Saarland University, Saarbruecken, Germany; 3 Department of Chemistry and Molecular Biology, University of Gothenburg, Gothenburg, Sweden; 4 Wallenberg Centre for Molecular and Translational Medicine, University of Gothenburg, Gothenburg, Sweden; 5 Faculty of Medicine, Saarland University, Homburg, Germany

**Keywords:** Glycan, Glycobiology, IUPAC-condensed, SMILES, Grammar

## Abstract

Glycans are important polysaccharides on cellular surfaces that are bound to glycoproteins and glycolipids. These are one of the most common post-translational modifications of proteins in eukaryotic cells. They play important roles in protein folding, cell-cell interactions, and other extracellular processes. Changes in glycan structures may influence the course of different diseases, such as infections or cancer. Glycans are commonly represented using the IUPAC-condensed notation. IUPAC-condensed is a textual representation of glycans operating on the same topological level as the Symbol Nomenclature for Glycans (SNFG) that assigns colored, geometrical shapes to the main monomers. These symbols are then connected in tree-like structures, visualizing the glycan structure on a topological level. Yet for a representation on the atomic level, notations such as SMILES should be used. To our knowledge, there is no easy-to-use, general, open-source, and offline tool to convert the IUPAC-condensed notation to SMILES. Here, we present the open-access Python package GlyLES for the generalizable generation of SMILES representations out of IUPAC-condensed representations. GlyLES uses a grammar to read in the monomer tree from the IUPAC-condensed notation. From this tree, the tool can compute the atomic structures of each monomer based on their IUPAC-condensed descriptions. In the last step, it merges all monomers into the atomic structure of a glycan in the SMILES notation. GlyLES is the first package that allows conversion from the IUPAC-condensed notation of glycans to SMILES strings. This may have multiple applications, including straightforward visualization, substructure search, molecular modeling and docking, and a new featurization strategy for machine-learning algorithms. GlyLES is available at https://github.com/kalininalab/GlyLES.

**Supplementary Information:**

The online version contains supplementary material available at 10.1186/s13321-023-00704-0.

## Introduction

### Glycans

In all kingdoms of life, cells, proteins, lipids, and other biological entities are often wrapped in chains of complex carbohydrates, known as glycans. Assembled by dedicated enzymes, glycosyltransferases, glycans form linear or branched sequences from a pool of monosaccharides that is to some degree specific to each species. The specific glycan sequence that is attached to a macromolecule modulates its properties, such as structure, stability, or function. This fact, together with glycan’s diversity, makes glycans key in modulating functionality in most fundamental physiological processes [1]. For example, the lack of core fucosylation of the glycan attached to the antibody leads to increased antibody potency [2], or upregulation of sialyl-Lewis X structures facilitating the interaction with selectin proteins in cancer metastasis [3].

In fact, the modulation of protein binding is in some ways most sensitive to subtle changes in glycan chemistry. Glycan-binding proteins, or lectins, have evolved to recognize the exact shape and chemistry of particular glycan substructures or motifs. Witnessed naturally in viral infection, for example in the form of interaction of viral proteins with host glycan receptors [4], or the innate immune system [5], this property is also widely used in the laboratory and clinic, for enriching, visualizing, and targeting cells or proteins with characteristic glycan structures [6, 7]. Changes in glycan chemistry have also been observed in historic evolution (e.g., loss of functional groups such as the *N*-glycolyl group in humans [8]), as well as in contemporaneous evolution, in the form of the arms race in glycan-mediated host-microbe interactions [9, 10].

### Terminology

First, we introduce some terms specific for this work. When talking about glycans, we refer to any natural carbohydrate structure that has a tree-like topology, which is most common in nature.

In total, GlyLES can process 59 monosaccharides (Additional file 1: Table S1) which includes all monosaccharides that cannot be split further into a monosaccharide and a modification based on the IUPAC-condensed string (e.g., 6dTal can be split as 6d + Tal). In this paper, we will use the terms monosaccharide and monomer interchangeably. The naming of monosaccharides can be ambiguous, e.g., AraHex is the same monosaccharide as Glc. Therefore, 2dAraHex practically does not occur in glycochemistry, but 2dGlc does. GlyLES does not perform semantical checks on the input, which is further discussed in the section on Features and Limitations, and compute the same SMILES string for both, 2dAraHex and 2dGlc, namely OC[C@H]1OC(O)C[C@@H](O)[C@@H]1O.

Modifications are small chemical moieties that can be attached to monosaccharides. One exception from this are Xd modifications where the carbon X is dehydroxylated and therefore nothing is attached but removed. There are cases when modifications are “undetachable”. This means, that a certain carbon atom cannot take any or another modification. An example of this is 2dDig. Digitose has no hydroxy group bound to carbon 2, so this group cannot be dehydroxylated.

The lists of monomers (Additional file 1: Table S1) and modifications (Additional file 2: Table S2) are mainly assembled based on the list of glycans in Glycowork [11]. Additionally, we extended the collection with the monosaccharides and modifications from the CSDB/SNFG Structure Editor [12].

### SMILES

SMILES (Simplified Molecular-Input Line-Entry System) is a widely accepted standard for representing any chemical molecule as an ASCII string, in which non-hydrogen (heavy) atoms are encoded with their chemical symbols, and a grammar is introduced for representing covalent bonds of different nature [13]. Whereas there are tools for translating the standard IUPAC to SMILES, e.g., OPSIN [14], there are no such tools for the IUPAC-condensed notation of glycans. In this work, we aim to bridge this gap by introducing GlyLES, a Python-based open-source package, that given an IUPAC-condensed string for a glycan outputs the corresponding SMILES string.

### Overview of different glycan notations

The structure of a glycan can be very complex due to many monosaccharides (59 in S1), extraordinarily many branching structures with different linkages, and a countless number of modifications that can be attached to any monosaccharide [15], particularly in bacteria.

Glycans are traditionally represented using a so-called *IUPAC-condensed notation*, which is a textual representation of glycans and a basis for SNFG images [16, 17] (Fig. 2a). The IUPAC-condensed notation differs from the standard IUPAC, as illustrated below by an example of $\alpha$-D-mannopyranose. In IUPAC, $\alpha$-D-mannpyranose has the description

$${(3{\text{S}},4{\text{S}},5{\text{S}},6{\text{R}}){\text{-}}6{\text{-}}({\text{hydroxymethyl}}){\text{oxane-}}2,3,4,5{\text{-tetrol}},} $$

or, following the IUPAC recommendations for Carbohydrates [15],

$$\begin{aligned} \texttt {a-D-mannopyranose,} \end{aligned}$$

whereas the IUPAC-condensed name is

$$\begin{aligned} \texttt {Man.} \end{aligned}$$

While this type of token-based notation has numerous advantages, including human readability and compactness, chemical similarities between monomers and substructures may occasionally be obscured. However, such similarities can substantially influence the biochemical properties of the resulting glycan. Examples include the binding specificity of the glycan-binding protein WGA [18]. In the IUPAC-condensed notation, its binding specificity (GlcNAc, GalNAc, Neu5Ac, MurNAc) seems very broad. Yet, on a chemical level, WGA specifically binds to *N*-acetyl moieties shared by these monomers. Other examples include lectins, such as MAL-I binding to negatively charged glycans [18], where the negative charge can be provided by both sulfate groups or sialic acids. Thus, an increased resolution in glycan notation can provide more general biological insights.

Besides the IUPAC-condensed notation, there are other glycan-specific notations. As discussed, IUPAC-condensed is a human-readable notation that is very hard to read by machines. Opposed to this, WURCS is an atom-focused, machine-readable notation for glycans [19, 20]. For the example of $\alpha$-D-mannopyranose, the WURCS 2.0 representations is

$$\begin{aligned} \texttt {WURCS=2.0/1,1,0/[a1122h-1x\_1-5]/1/.} \end{aligned}$$

WURCS has been developed to describe glycans in a single line to be used as a Uniform Resource Identifier (URI) in Semantic Web.

A notation bridging human-readability and machine-readability is the CSDB Linear Notation [21, 22]. It is also used as a URI in the Carbohydrate Structure Database but preserves the advantages of the IUPAC-condensed notation of being human-readable and operating on a topological level. The exemplary $\alpha$-D-mannopyranose is denoted as

$$\begin{aligned} \texttt {aDManp.} \end{aligned}$$

Lastly, a notation we will use in this work is the Symbol Nomenclature for Glycans (SNFG) [16, 17]. It is a graphical visualization of the topological structure of glycans by assigning colored, geometric shapes to the most common monosaccharides. These shapes are then connected by lines representing bonds between the individual monomers. Additionally, the bonds are labeled with respect to the bond type - whether it is an $\alpha$-glycosidic bond or a $\beta$-glycosidic bond - and to which atom of the higher-level monomer (in the glycan tree) the lower-level monomer is attached. An example of this representation can be seen in Fig. 2a and a complete overview with more examples is provided in [23].

### IUPAC-condensed notation of glycans

The IUPAC-condensed notation of glycans is one of the most commonly used notations in publications, for example, [11, 18], databases, where examples are [24–27], and other resources. Its wide usage is because of the compact descriptions of glycans and the human-readability of the notation [28].

This notation is a specialized form of the general IUPAC notation that is used to describe other organic molecules in a standardized way [29]. It is specifically suited for glycans and their variability in structure and added modifications in a compact form.

For simplicity, from now on we will refer to the IUPAC-condensed notation using the term “IUPAC” as the original IUPAC notation does not play a role in the presented work.

Glycans have a complex, yet regular, structure, especially when described using the IUPAC notation. For example,

$$\texttt {Man(a1-4)Man(a1-4)Man(a1-4)Man}$$

is a simple chain of four mannopyranose monosaccharides connected with 1–4 alpha-*O*-glycosidic bonds. Important to note is that the root of the glycan tree is always the last monosaccharide in an IUPAC formula. The chain grows from the right by prepending elements like Man(a1–4), which corresponds to adding a new mannose to a leaf-monomer of the glycan. The branching of the trees is described by introducing the new branch into square brackets. For example,

$$ \texttt {Man(a1-4)Man(a1-3)[Man(a1-4)Man(a1-4)]Man(a1-4)Man} $$

is a tree of mannopyranoses that has two monosaccharides before splitting into two chains with again two mannopyranoses each. The branching is put right in front of the monomer, where the root of the side branch is bound to the main strand. This can be extended to three chains of two monosaccharides, each bound to a single mannopyranose:

$$ \texttt {Man(a1-4)Man(a1-2)[Man(a1-4)Man(a1-3)][Man(a1-4)Man(a1-4)]Man.}$$

Modifications in IUPAC are directly annotated at the changed monosaccharide. The annotation is often done by first naming the position of the carbon atom where the monomer is modified. Then, an abbreviation for this group is provided. So, for example, Man3S describes a mannose with a sulfur group attached to the third carbon atom. The number can also be dropped in case of a modification occurring on a standard position, as in GalNAc where an acetamide group is attached to the second carbon atom of galactopyranose. There are other possibilities to denote modifications, but they are not addressed in this paper.

In the chemistry of glycans, modifications can be attached to any oxygen, nitrogen, or carbon atom in a monosaccharide (e.g., Man4S, Man4P, or ManNS), and they can be combined in any way (e.g., Man3S4P). The only restriction is that one cannot attach two modifications to the same atom (e.g., Man2S2P is not possible).

### Compilers

In computer science, compilers translate source code into machine code. Source code is written in high-level, human-readable languages such as Java, C/C++, or Python. Machine code comprises a set of commands executed by the processor, such as adding the content of two registers or moving the content from one register to another. In short, compilers translate one notation, source code, into another notation, machine code, where both notations describe the same computer program. We take the idea of compilers and apply it to the IUPAC notation to translate it to SMILES. IUPAC notations of molecules are more human-readable than SMILES, especially for molecules of the size of glycans that might contain hundreds of heavy atoms. Therefore, we can see the IUPAC notation as a source code composed of so-called tokens that are combined following rules of the notation, which jointly constitute a *grammar* [30].

Aside from the grammar, a compiler comprises a *lexer* and a *parser* that performs the conversion from human-readable programming languages into machine code based on the grammar. Or, in GlyLES, from IUPAC representations into SMILES. The lexer reads the source code, the IUPAC representations, and recognizes tokens in the input. Tokens are single or multiple characters, such as def, lambda, or : in Python. Examples from the IUPAC notation for glycans are [ or Man. The output of a lexer is a so-called token stream that is processed by a parser to generate a so-called *abstract syntax tree (AST)*. The AST represents the internal structure of the input. Generating a lexer and parser based on some grammar can be automated using tools such as ANTLR [31].

**Fig. 1:**
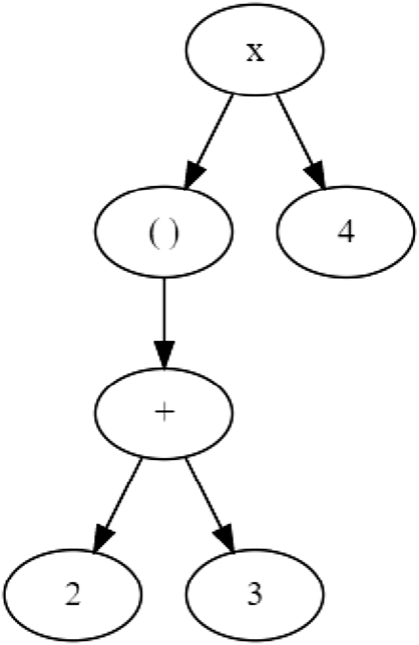
Example of an abstract syntax tree (AST) for the arithmetic expression $(2+3)\times 4$

An example of this process can be seen when parsing $(2+3)\times 4$ using simple arithmetic rules. These rules from analysis correspond to the parser rules of a compiler, whereas identifying the individual symbols as numbers, brackets, and arithmetic signs is what the lexer does. The output of the lexer, i.e. the token stream, is

$$\begin{aligned} \texttt {( 2 + 3 ) x 4} \end{aligned}$$

and the result of the parser, the AST, can be seen in Fig. 1.

### Previous work

To the best of our knowledge, there is no tool able to convert IUPAC notations of glycans into SMILES that works offline for arbitrary glycans in an automated fashion. Even in the century of online services, web-APIs, or WSDL, we still believe tools working offline to be an advantage, as offline tools are easier to maintain and develop, especially for open-source projects such as GlyLES. Additionally, online tools cannot be included in pipelines easily. The fact that GlyLES is fully written in Python with very few dependencies provides an additional advantage for using it in automated pipelines, too.

Alternatives can be divided into two groups: Databases that store information on glycans, including their SMILES string, from which mappings between IUPAC and SMILES strings can be extracted; and online tools converting IUPAC to SMILES.

Several databases, such as Glyco3D [26], GlyCosmos [27], and GlyGen [32], are specific for glycans and provide IUPAC strings among other notations. PubChem [24] also contains some glycans which are annotated with both IUPAC and SMILES. Yet all these databases are static and cannot be used to retrieve the SMILES notation for a not yet stored glycan, which is not uncommon given the large variety of glycan structures. Further, obtaining SMILES notation of a glycan motif can be cumbersome with this approach, as most databases only store full glycans, not motifs, and it is not human-readable which exact part of the SMILES string corresponds to which substructure in the IUPAC notation.

Among specialized online tools, REStLESS [33] and the “CSDB/SNFG structure editor” [12] are both based on the Carbohydrates Structure Database [34]. REStLESS uses an IUPAC-condensed-like notation as a starting point when converting to SMILES. Neither of the tools is open-source or available for offline use. REStLESS may require pre-processing of input sequences, as they do not work on standard IUPAC-condensed glycans. Therefore, REStLESS cannot be seen as easy-to-use. The “CSDB/SNFG structure editor” has an online interface where one can build the glycan of choice and copy-paste the SMILES string, which is not workable to do in an automated fashion.

In this work, we present a tool that can convert arbitrary glycans from an IUPAC representation into SMILES representation that can further be used to build atomic graphs for the glycans.

## Implementation

The overall structure of the package comprises three major steps. First, we read in an IUPAC string and parse it into an AST based on a grammar. Second, we compute a SMILES string for each monomer in the nodes of the AST. The grammar for this can be found in the GitHub repository as well (https://github.com/kalininalab/GlyLES/blob/main/glyles/grammar/Glycan.g4) This includes adding all modifications to the monosaccharide and storing the monomers as objects in the nodes of the AST. Finally, the tree is resolved into a single SMILES string representing the input IUPAC-encoded glycan in the SMILES notation. We will have a closer look at all these steps in the following sections.

### Step 1: Parsing IUPAC notation into AST

The IUPAC notation has a very regular structure to describe glycans. Knowing the internal structure of the IUPAC nomenclature for glycans, one can implement a grammar recognizing this general structure. Based on the grammar, there are tools such as ANTLR generating the lexer and parser for some grammar.

As described in the section about Compilers, the output of a parser is an AST, which is a tree of the tokens in the input. For a properly defined grammar, the AST is very similar to the structure of the glycan, as both structures are trees. Here, we want to emphasize that GlyLES is developed for glycans that are rooted trees, and cycles or unrooted trees are currently beyond the scope of GlyLES. For more information, see the section on Features and Limitations below. Thus, the root monomer of the glycan corresponds to the root node of the AST. The leaf monosaccharides in the glycan can be found in the leaves of the AST. This has the benefit that we do not have to convert the output of the parser into some other structure. but can take it as it is.

**Fig. 2:**
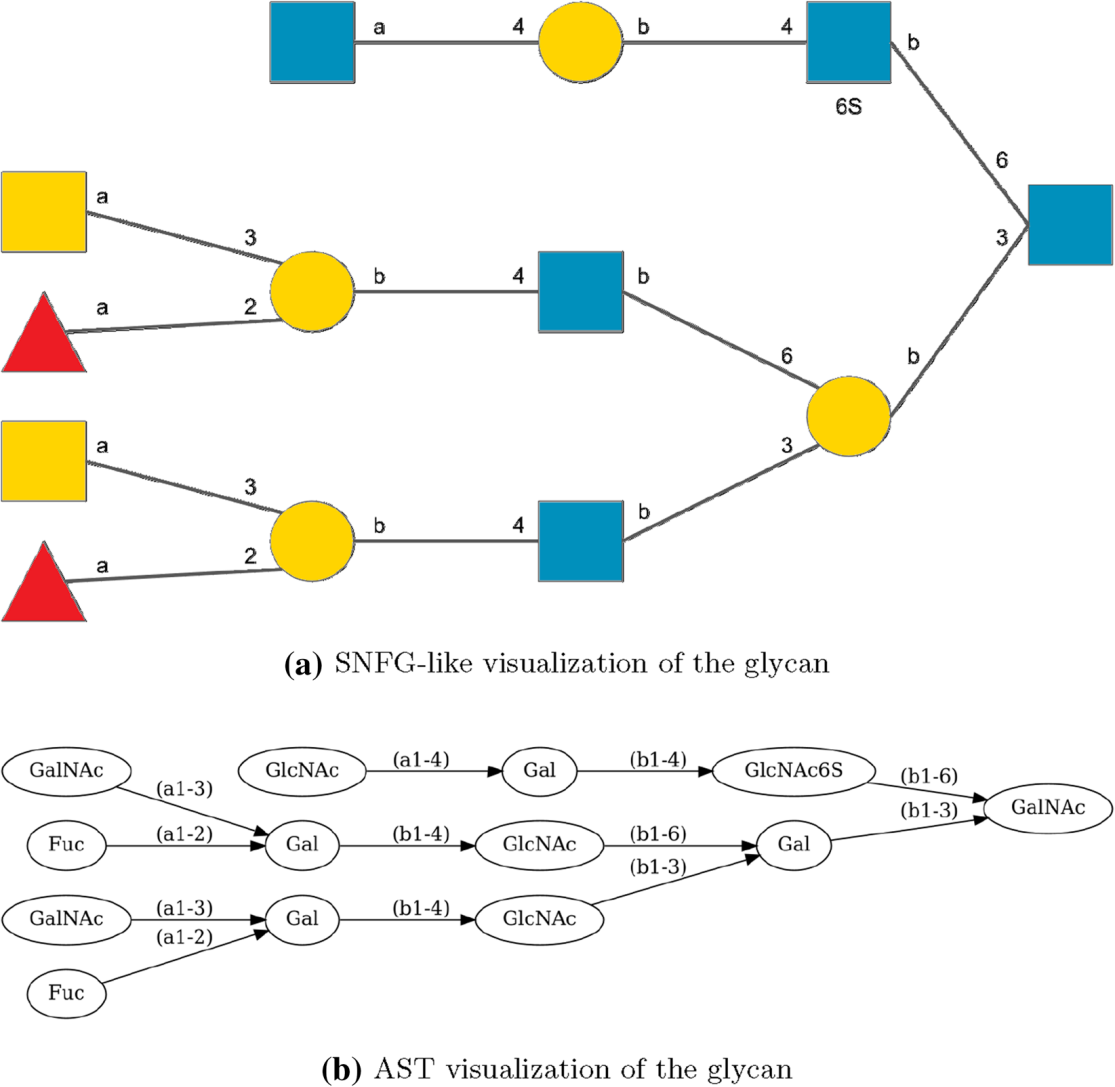
**a** SNFG image representation of the glycan in the implementation example. **b** AST representation of the glycan in the implementation example. In every node, we provide the IUPAC description for the single monosaccharide, including all its modifications as the label

For example, the AST for a glycan from human gastric mucins with an additional sulfate attached to a GlcNAc is very similar to the actual glycan structure (Fig. 2a and b) [35]. The corresponding IUPAC string is
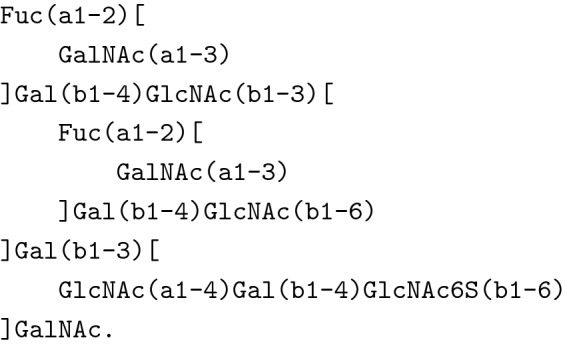


We formatted the IUPAC with line-breaks wherever a new branch is introduced into the parent strand to better understand the structure.

### Step 2: Applying modifications

After parsing the IUPAC representation into an AST, all monosaccharides of the glycan are stored in the nodes of the tree. Now, we have to compute the SMILES string for the monomer in each node, including all its modifications.

In this package, applying modifications to monosaccharides is done by the grammar using another helpful property of the IUPAC notation. From the example of Gal(a1–6)ManNAc4S(a1–4)Gal, we can see that everything between the two bonds belongs to the mannose. Thus, the information on how to modify the SMILES description of the mannose can be stored directly in the node representing the mannose in the AST. The information on how to apply modifications to monomers is provided in a rule in the grammar, and modifications are given as tokens. This has the effect that every monosaccharide node in the AST stores information about the modifications to be applied.

Once the AST is constructed, we can iterate over the nodes and generate a SMILES string for every monomer, including its modifications. This is done by first identifying the monosaccharide in a node and whether it is in the alpha or beta enantiomeric form and creating its RDKit representation. To apply a modification, we replace all lost atoms in a monosaccharide the modification binds to by a single placeholder atom. In parallel, we look up the name of the modification in a map from modification names to SMILES strings. Then, the monosaccharide with the placeholder is converted to a SMILES string, the placeholder is replaced with the SMILES string of the modification, and the SMILES is converted back into an RDKit molecule again.

**Fig. 3:**
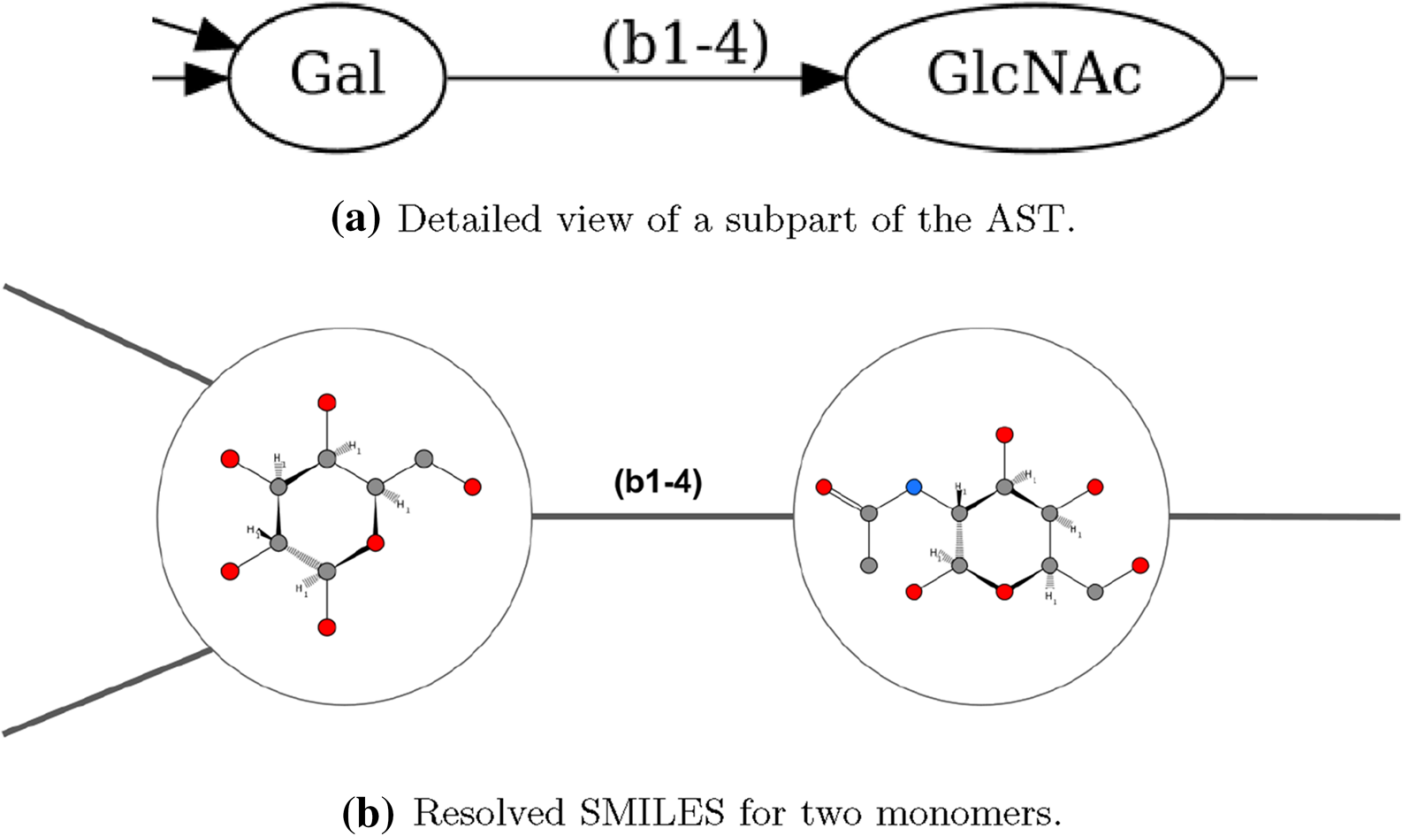
Adding modifications. **a** Zoom into the AST displaying the branch between Gal and GlcNAc in the running example. **b** The corresponding part of the AST with monosaccharides resolved to the atomic level and included modifications. Chemical structure visualization done with https://smarts.plus/ [36]

In the example from the previous step, we zoomed in on a connection of two monomers to show the procedure of step 2 in more detail (see Fig. 3).

### Step 3: Connecting monomers

In the last step, we combine all the monomers in the nodes into a single SMILES string representing the entire glycan. This is done similarly to how we added the modifications to the monosaccharides. First, we identify the position where a child monomer binds, replace that oxygen (works similarly for nitrogens, while for carbons we replace a hydrogen atom) with a placeholder atom, generate a SMILES string and replace the placeholder atom with the SMILES string of the child monomer. This is done recursively until we put together the SMILES string for the root monosaccharide, which is the SMILES string for the represented glycan.

The result of this operation for the example in Fig. 3a and b is
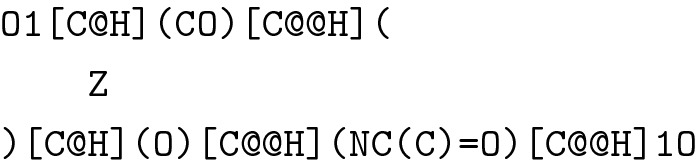


for the GlcNAc b where the SMILES string for Gal b will replace the Z. Gal b is translated into
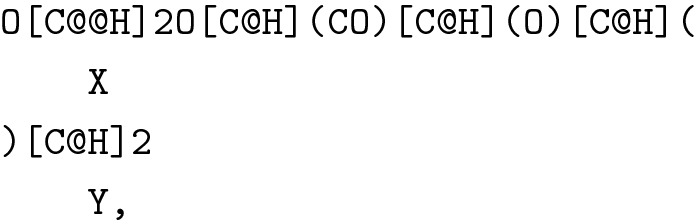


where X and Y will be replaced by the SMILES strings for GalNAc a and Fuc a from the parts of the tree that are not shown in Fig. 3a and b.

If we plug in the part for Gal b, we get
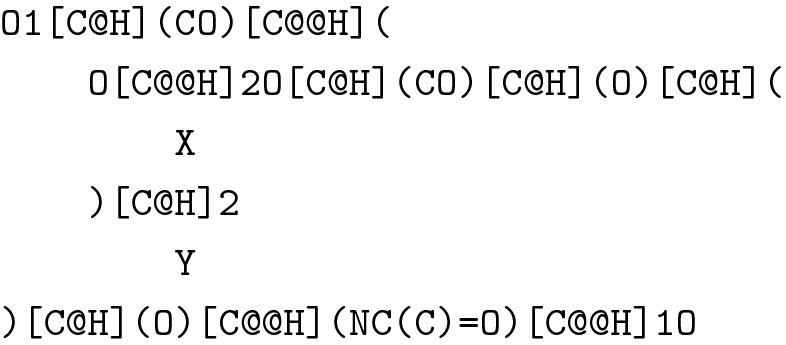


showing the principle of how the SMILES string of the monosaccharides are plugged into each other.

**Fig. 4:**
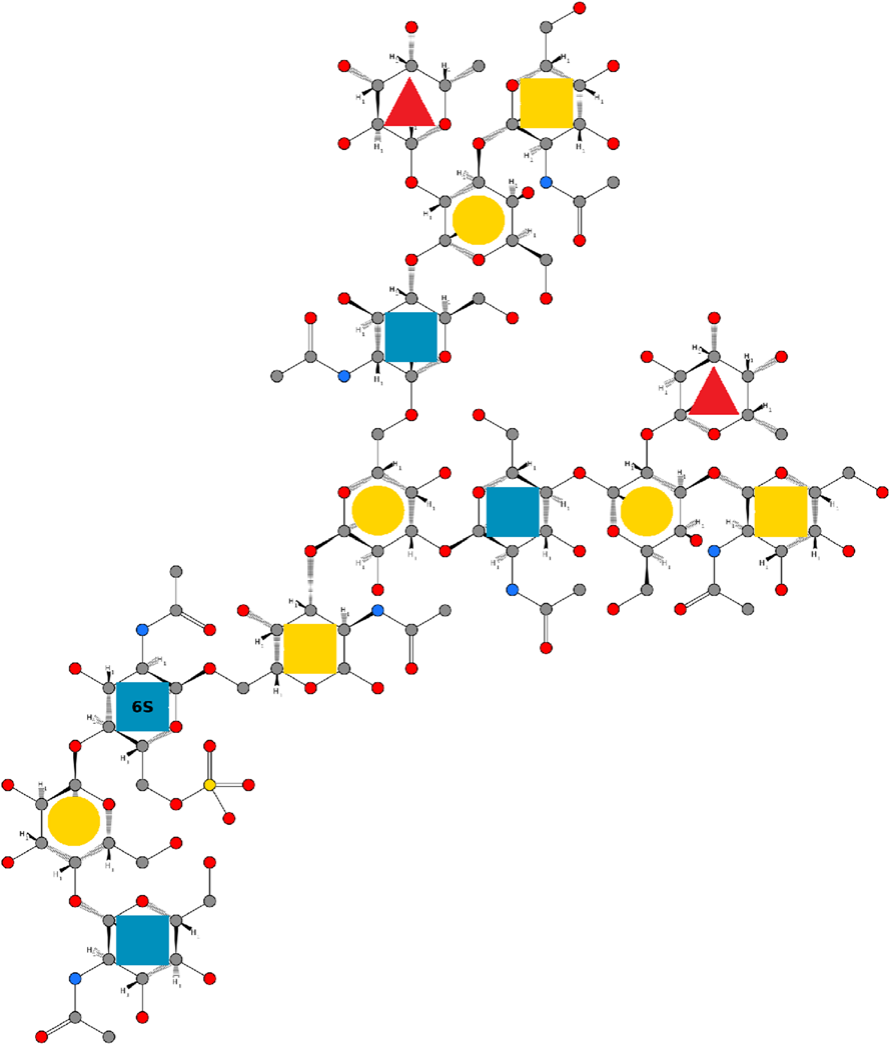
Visualization of the example glycan on an atomic level with the help of the GlyLES package. Chemical structure visualization done with https://smarts.plus/ [36]

## Results and discussion

GlyLES has been tested using handcrafted tests aided by fuzzy tests using the pytest framework [37]. The fuzzy tests generate structures and check if the parsed AST is correct and stores all information correctly. Additionally, we scanned the entire PubChem database to collect a large set of $\sim$ 8000 examples to test if the tool works under real-world conditions. All these tests are included in the published code on GitHub (https://github.com/kalininalab/GlyLES). Comprehensive lists of all implemented monosaccharides and modifications are provided as Additional file.

The tool aims to convert any glycan with any combination of modifications given by its IUPAC-condensed notation into a SMILES string. GlyLES can do most of this as a Python package that is easy to use, offline, and open-source. The current implementation can process the majority of glycans, yet some rare monosaccharides, modifications, and structures are not yet implemented. A more extensive list of features and limitations is given in the corresponding section below.

For the subset of glycans in the Glycowork dataset [11] that have a complete structural description without wildcard connections between monosaccharides ($\sim$24,000 structures), GlyLES can convert $\sim$99% of the glycans. In this dataset, there are no labels and we can only test if the output of the tool is a valid, organic molecule. We also extracted pairs of IUPAC-condensed and SMILES representations for glycan from the PubChem database to test if the tool also produces SMILES string representing the true molecules ($\sim$8000 structures). In all cases, GlyLES reconstructed the atomic structure that is stored within the compound entry in the PubChem database.

For example, the SMILES string for the molecule in Fig. 2a is
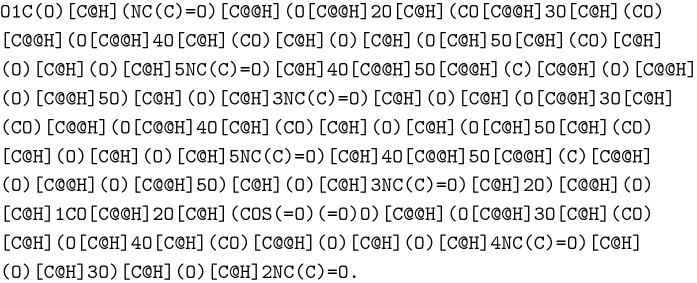


Figure 4 visualizes this on an atomic level and highlights the single monomers with their symbols from SNFG.

### Runtime

To estimate the average time that GlyLES needs to convert a glycan, we ran a timed test on a subset of our test glycans. We took those glycans we had the SMILES strings for, to be sure GlyLES does not raise an error and the output will be correct. Error-free output is important because raising an error costs additional time and would distort the result. The tested glycans differ largely in their size and shape to get a better average estimate of the runtime. Overall, we converted a list of 11,951 IUPAC names of glycans into a list of SMILES strings. This process lasts $\sim$232 s, which results in an average time per glycan of $\sim$19 milliseconds. This measurement was conducted on a single 11th Gen Intel Core i7–11,700 with 2.5 GHz.

### Features and limitations

Building on the explained conversion, GlyLES can convert glycans assembled in lists, from a generator, or from a file, and print the SMILES strings to the command line, return them as a generator, or store them in a file. The structural features that can be converted are not limited to the simple cases presented above, but GlyLES can also convert glycans containing inline phosphates and sulfates as well as sequences of modifications, where a modification of a monomer is modified itself.

Apart from just converting glycans from IUPAC into SMILES, there are many other tasks one can use GlyLES for. Given a glycan in the IUPAC notation, GlyLES can visualize glycans using the DOT-language [38] or as an SNFG-like image [16, 17]. Furthermore, GlyLES can count substructures (one or more monomers) and functional groups in the parsed and converted glycans based on the computed SMILES string.

Examples for all these tasks can be found on the readthedocs-page of the project (https://glyles.readthedocs.io).

However, there are some limitations to the input as well. Some monosaccharides, modifications, and structural features of glycans are so rare that they are not yet implemented. These limitations include all monomers not listed in Additional file 1: Table S1 and all modifications not listed in Additional file 2: Table S2. Apart from these easy-to-mitigate limitations, there are more difficult ones such as inner repeats (Man(a1–4){Gal(a1–4)}5Tal), modifications replacing more than one non-hydrogen atom, cyclic and unrooted glycans, non-sugar components in the backbone, and structural uncertainties, such as wildcard bonds. Structural uncertainties are intrinsically impossible to resolve since if the structure is not known, one cannot generate a SMILES string. One could generate all possibilities but with an increasing number of uncertainties, the number of possibilities explodes. For example, a glycan with *n* wildcard bonds like (?1-?) would have $\sim$ $8^n$ different SMILES strings (assuming alpha or beta configuration for the first ? and 4 for the second ?).

Additionally, we provide the interested reader with a table of errors thrown by GlyLES, in case GlyLES hits its limitations (Additional file 3: Table S3). This table only contains glycan-related errors, there are more errors related to input validation (e.g., check if a provided file path is valid).

So far, GlyLES does not perform semantical checks of the input. This means, if the input to GlyLES is a valid glycan, GlyLES returns the atomic structure in SMILES notation. If the input is an invalid glycan, the result can be an error printed and an empty string is returned.

## Conclusion

To our knowledge, GlyLES is the first free, offline, and open-source tool to convert glycan representations from IUPAC-condensed notation to SMILES. It can compute SMILES representations of glycans in research projects where glycans need to be represented on an atomic level. Such applications include, but are not limited to, glycan visualization, substructure search and comparison, molecular modeling, docking, and molecular dynamics, or featurization for machine learning and deep learning models. GlyLES is more flexible, extendable, and easier to maintain than databases and conversion tools on top of databases that are primarily used so far. It is also more generalizable and efficient than databases, as all glycans are computed during runtime and it is not necessary to store anything.

## Availability and requirements

- **Project Name:** GlyLES
- **Project home page:** https://github.com/kalininalab/GlyLES
- **Operating system(s)** Platform independent
- **Programming language:** Python
- **Other requirements:** None
- **License:** MIT License
- **Any restriction to use by non-academics:** No restrictions

## Supplementary Information

**Additional file 1: Table S1.** List of parsable monomers.

**Additional file 2: Table S2.** List of parsable modifications.

**Additional file 3: Table S3.** List of error messages of GlyLES.

## Data Availability

Archived version under the project home page, see the Availability and Requirements section for further details.

## Abbreviations

ANTLR: Another Tool for Language Recognition
ASCII: American Standard Code for Information Interchange
AST: Abstract syntax tree
Fuc: Fucose
Gal: Galactose
GalNAc: N-Acetyl-Galactosamine
GlcNAc: N-Acetyl-Glucosamine
GlcNAc6S: 6-Sulfo-N-Acetyl-Glucosamine
IUPAC: International Union of Pure and Applied Chemistry
MAL-I: Maackia amurensis-I
Man: Mannose
MurNAc: N-Acetyl Muramic Acid
Neu5Ac: N-Acetyl Neuraminic Acid
SMILES: Simplified molecular-input line-entry system
SNFG: Symbol Nomenclature for Glycans
Tal: Talose
WGA: Wheat germ agglutinin
