# Supplementary material for: GlyLES: Grammar-based Parsing of Glycans from IUPAC-condensed to SMILES
Source: J Cheminform. 2023 Mar 23;15:37. doi: 10.1186/s13321-023-00704-0 (PMC10035253; doi:10.1186/s13321-023-00704-0)
Supplement: Supplementary file 3 — Additional file 3: Table S3. List of error messages of GlyLES. [file 13321_2023_704_MOESM3_ESM.pdf]

Error messages and explanations for the GlyLES Python Package

Message (concrete form might vary as error message depends on the input)	Explanation of why the input is wrong (not what to correct)	Command
Anhydro functional groups should have exactly two numbers: X,Y-Anhydro-…	An anhydro-groups should be given with two indices indicating which oxygens to react.	convert("3-Anhydro-Gal")
There is no oxygen, nitrogen, and hydrogen attached to C<i>! No functional group can be attached there!	The IUPAC-condensed string intends to attach a functional group to some position that cannot take that kind of modification.	
GlyLES can only link monomers with N-glycosidic linkages and O-glycosidic linkages.	Linking a monosaccharide to a carbon of another monosaccharide that cannot take a linkage at that position.	convert(“Man(a1-2)Dig”)
Multiple or no options for oxygen (or other atom type) found.	Linking another monomer or attaching a monomer to a carbon that does not exist.	convert(“Man(a11-2)Gal”)
C4={t6} defined modifications for carbon atoms that are not modifiable.	In these special carbon chains, the modification is defined invalidly. There cannot be a trans C-C double bond at C6 if there are only 4 carbons in that chain.	convert(“C4={t6}Gal”)
Glycan cannot be parsed: line 1:19 no viable alternative at input '{Gal(a1-2)Man(b1-3)]'	IUPAC-condensed string given that is either invalid or includes features not yet implemented.	Glycan(“Gal(?1-?)Man”)
